# Ion‐Exchange‐Driven Ternary Organic Neuromorphic Devices with High Efficiency and Stability

**DOI:** 10.1002/advs.77486

**Published:** 2026-09-08

**Authors:** Sungmin Lee, Vivek Pratap Singh, Hyeonryul Lee, Young‐Yong Kim, Yong‐Ryun Jo, Changhoon Lee, Ji Hoon Shim, Namsoo Lim, Guanjie Wang, Junyeong Sung, Dongyeop Yang, Zhongwu Wang, Liqiang Li, Chandran Balamurugan, Sooncheol Kwon

**Affiliations:** ^1^ Department of Energy and Materials Engineering Dongguk University‐Seoul Seoul Republic of Korea; ^2^ Department of Advanced Battery Convergence Engineering Dongguk University‐Seoul Seoul Republic of Korea; ^3^ Department of Electronics and Communication Engineering Maulana Azad National Institute of Technology (MANIT) Bhopal Madhya Pradesh India; ^4^ Pohang Accelerator Laboratory Pohang University of Science and Technology Pohang Republic of Korea; ^5^ GIST Advanced Institute of Instrumental Analysis (GAIA) Electron Microscopy Laboratory Gwangju Institute of Science and Technology Gwangju Republic of Korea; ^6^ Max Planck POSTECH Center for Complex Phase of Materials Pohang University of Science and Technology Pohang Republic of Korea; ^7^ Department of Chemistry Pohang University of Science and Technology Pohang Republic of Korea; ^8^ Research Institute of Molecular Alchemy Gyeongsang National University, Jinju South Gyeongsang Republic of Korea; ^9^ State Key Laboratory of Advanced Materials for Intelligent Sensing & Key Laboratory of Organic Integrated Circuit Ministry of Education & Tianjin Key Laboratory of Molecular Optoelectronic Sciences Department of Chemistry Institute of Molecular Aggregation Science Tianjin University Tianjin China

**Keywords:** electrochemical doping, In situ observation, ion exchange, organic neuromorphic device, ternary blend system

## Abstract

Organic neuromorphic platforms based on top‐gate ionic‐liquid architectures rely on precise electrochemical doping at low operating voltages; however, intrinsically unfavorable polymer‐ion interfaces often lead to unstable doping, elevated operating voltages, and poor device‐to‐device uniformity. Herein, we present a solution‐processed ternary organic blend system composed of organic semiconductors and a binary ionic‐liquid system, in which spontaneous ion exchange within the active layer establishes a stabilized ionic environment and facilitates anion access into semicrystalline domains. In situ X‐ray and Raman spectroscopy measurements reveal that this stabilized ionic matrix enables efficient anion penetration and electrochemical doping even under short gate pulses. The resultant ternary‐blend devices operate under a low gate voltage (V_GS_ = −3.5 V) while effectively suppressing rapid de‐doping after bias removal. Notably, the ternary organic blend system exhibits consistent and enhanced electrochemical doping behavior across semiconducting polymers with fundamentally different backbone chemistries, ranging from thiophene‐based donor polymers to donor‐acceptor conjugated systems. In particular, the devices exhibit remarkable electrical and neuromorphic performance, including long retention (∼25h), stable operation over ∼10^3^ sequential pulses, and reliable synaptic characteristics under various stimuli. This ternary strategy enables high‐reliability organic neuromorphic systems, achieving 97.98% recognition accuracy in CNN‐based MNIST classification.

## Graphical abstract

Spontaneous ion exchange in a ternary polymer–ionic liquid blend disrupts the phase‐separated ionic domains of the binary film and creates a more homogeneous polymer–ion environment. This ionically reorganized matrix enables stable and efficient electrochemical doping, leading to ion‐gel‐gated organic electrochemical transistors with enhanced electrical and synaptic performance.

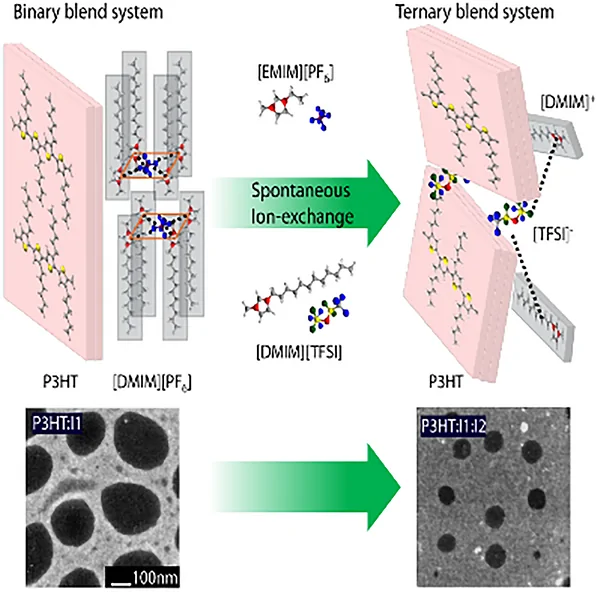

## 1 Introduction

Electrochemical neuromorphic devices based on conjugated polymers (CPs) have attracted growing interest as energy‐ and cost‐efficient platforms for information processing, as ionic doping enables low‐voltage operation and continuous modulation of conductance in the CPs [1, 2, 3, 4, 5]. This capability also aligns with the recent shift toward physical neuromorphic computing, where intrinsic device dynamics are directly exploited for information processing [6, 7, 8, 9, 10]. Achieving such computing capabilities requires precise control of ionic dynamics together with stable and reproducible device performance. Ion‐gel‐gated organic electrochemical transistors (IG‐OECTs) offer a distinctive materials platform, as electronic and ionic transport are intrinsically coupled within solution‐processable polymer solids that are mechanically flexible, stretchable, and chemically tunable [11, 12, 13, 14, 15]. This coupled transport allows gradual and reversible conductance modulation through electrochemical doping, providing a direct means to emulate key synaptic functions such as short‐term plasticity (STP) and long‐term plasticity (LTP) including both potentiation and depression [16, 17, 18].

Despite these advantages, conventional CP‐based IG‐OECTs still suffer from fundamental limitations in achieving stable long‐term memory and reliable operation under repeated ionic gating. These challenges arise from the molecular incompatibility between nonpolar alkyl‐rich CPs and polar mobile ions, leading to shallow ion penetration, rapid back‐diffusion, and unstable electrochemical doping [19, 20, 21, 22, 23]. Although semicrystalline domains can suppress ion back‐diffusion and improve long‐term retention, their weakly solvating environment imposes a substantial energetic barrier to ion insertion [24]. Hence, the trade‐off relationship between limited ion accessibility and stable ionic retention significantly limits effective polymer‐ion interactions, resulting in poor synaptic retention as well as device‐to‐device uniformity.

One strategy to mitigate these issues is to incorporate ionic liquid (IL) into the CP matrix. However, conventional short‐alkyl‐chain ILs, owing to their high polarity, exhibit poor interfacial compatibility with alkyl‐rich CP films, leading to severe de‐wetting and morphological instability driven by surface‐energy mismatch. As a result, their use as blending additives in CP systems has been largely limited. Alternatively, introducing long alkyl chains into ILs reduces this mismatch, enabling molecular‐level electrochemical doping at low operating voltages, while preserving solution processability and scalability [25, 26]. However, strong cation‐anion electrostatic interactions still drive phase separation in CP:IL thin films, preventing a relatively stable and free anion environment and reproducible device operation. These limitations highlight the need for a fundamentally different blending strategy that weakens intrinsic cation‐anion coupling while promoting favorable ion‐polymer interactions throughout the bulk.

Recent efforts have sought to address these challenges by modifying the molecular structures of CPs. For instance, substituting conventional alkyl side chains with polar or ‐OH end groups (i.e., glycols) improve writing‐erasing characteristics in IG‐OECTs [27, 28]. However, such approaches often introduce excessive ion permeability, leading to ionic swelling and disruption of π‐π stacking, ultimately compromising structural integrity. In parallel, incorporating a cross‐linker into the CPs solution followed by post‐treatment has been reported to enhance the stability of IG‐OECTs [29, 30]. While crosslinking can suppress swelling and morphological degradation, it often reduces ionic accessibility and thus hinders deep ion injection into confined semicrystalline domains, limiting efficient volumetric electrochemical doping. As a result, current strategies remain unable to establish stable ionic control that enables both deep ion injection into the confined semicrystalline alkyl domains and suppressed de‐doping, thereby leaving a critical challenge in reliable IG‐OECT‐based neuromorphic platforms.

In this study, we introduce a ternary organic blend strategy that facilitates efficient and reliable organic neuromorphic operation. By incorporating a solid‐state IL (1‐dodecyl‐3‐methylimidazolium hexafluorophosphate, [DMIM]^+^[PF_6_]^−^, referred to here as I_1_) and a liquid‐state ionic liquid (1‐ethyl‐3‐methylimidazolium bis(trifluoromethylsulfonyl)imide [EMIM]^+^[TFSI]^−^, referred to here as I_2_) within a CP active layer, the distinct cation structures induce spontaneous ion exchange between [PF_6_]^−^ and [TFSI]^−^, creating a stabilized ionic environment in which [PF_6_]^−^ experiences weakened ion‐pair interactions. In situ grazing‐incidence wide‐angle X‐ray scattering (GIWAXS) and Raman spectroscopy directly reveal that this unique ionic state facilitates anion penetration into lamellar domains and induces anisotropic ion dynamics. This feature enables stable electrochemical doping under a low gate voltage (V_GS_ = −3.5 V) while suppressing rapid de‐doping after bias removal. As a result, the devices with the ternary blend system (CP:I_1_:I_2_) exhibit enhanced electrochemical stability, long‐term memory retention, and improved interfacial compatibility with ion‐gel gates, achieving a high on/off ratio (∼10^3^), extended retention (60% residual *I*_DS_ after 1500 s), and 97.98% accuracy of a convolutional neural network (CNN) based classification of the MNIST dataset.

## 2 Results

### 2.1 Design and Structural Stabilization of the Ternary Organic Blend System

**FIGURE 1:**
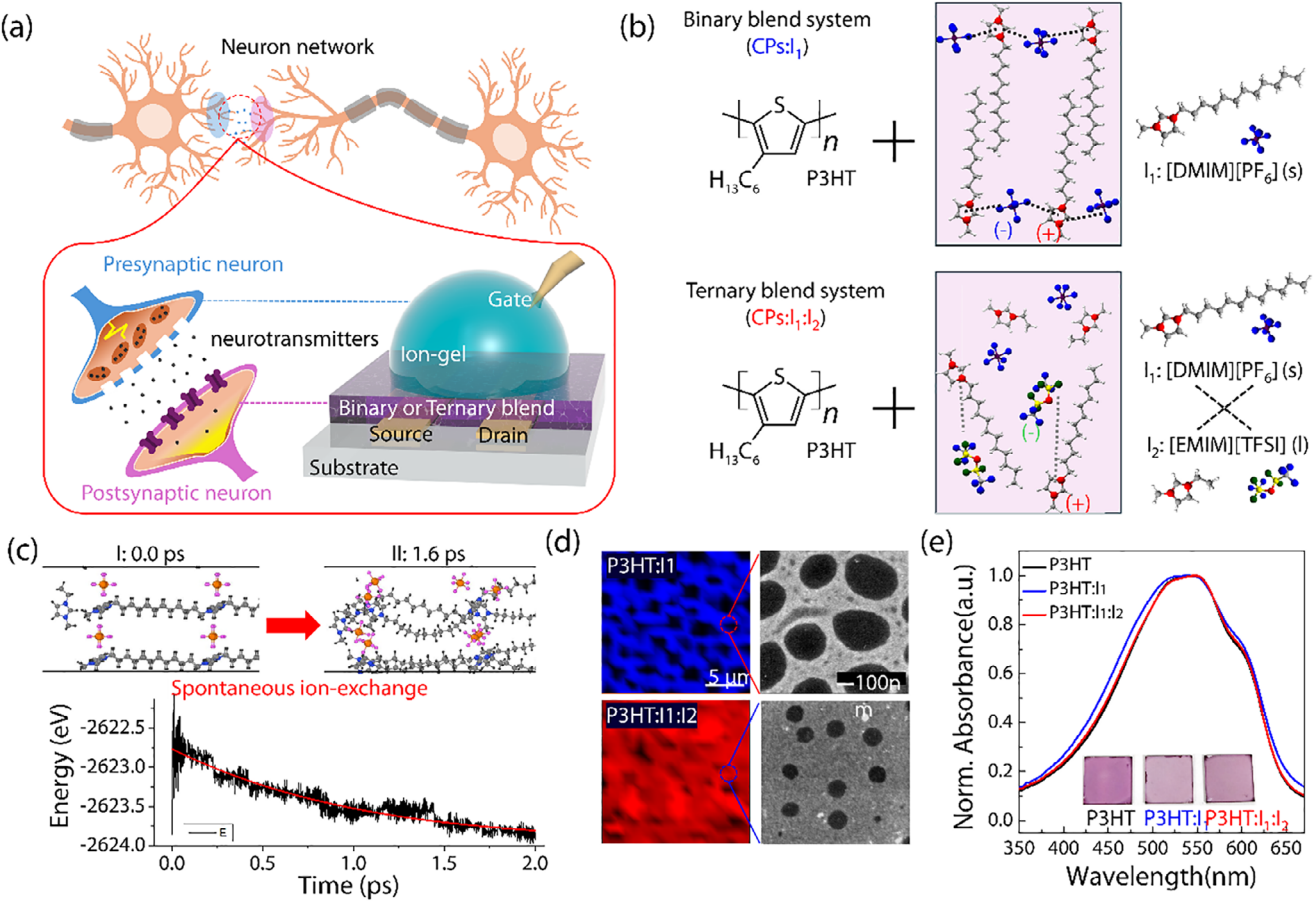
Device architecture of binary/ternary‐blend organic electrochemical transistors and their multi‐scale structural and optical characterization. (a) Schematic illustration comparing a biological neuron network and synapse with an ion‐gel‐gated organic electrochemical transistor (IG‐OECT). Configurations of IG‐OECT emulate the functionality of neuron with synapse. (b) Expected molecular environments of the binary blend system (P3HT:I_1_) and ternary blend system (P3HT:I_1_:I_2_), where I_1_ and I_2_ denote [DMIM]^+^[PF_6_]^−^, and [EMIM]^+^[TFSI]^−^, respectively. (c) DFT calculation of ion dynamic simulation and Gibbs free energy plot between I_1_ and I_2_ exchange process on the picosecond timescale. The initial state of ionic liquids at 0ps and final state of ion exchange are shown in I and II. (d) The mapping analysis of Raman spectroscopy after ion‐gel washing and Transmission electron microscopy (TEM) images of P3HT:I_1_ and P3HT:I_1_:I_2_. (e) Normalized UV‐Vis absorption spectra of three samples (P3HT, P3HT:I_1_ and P3HT:I_1_:I_2_) in thin‐film.

The schematic illustration describing the analogy between a biological synapse and an IG‐OECT is shown in Figure 1a. In the human brain, signal transmission and memory formation are governed by the migration of neurotransmitters between presynaptic and postsynaptic neurons. To emulate these synaptic functions, the IG‐OECT adopts a device architecture where the ion‐gel gate and the CP channel correspond to the presynaptic and postsynaptic neurons, respectively [14, 21, 31, 32]. We fabricated two types of devices, a binary blend system (P3HT:I_1_) and a ternary blend system (P3HT:I_1_:I_2_), to examine the effect of the blending strategy on the CP matrix (Figure 1b). We first hypothesized that, in the binary blend, the strong intrinsic ion pairing between the long‐alkyl‐chain imidazolium cation [DMIM]^+^ and [PF_6_]^−^ promotes ionic aggregation and phase separation from P3HT [33]. In contrast, the incorporation of I_2_ was expected to reorganize ion pairing through dynamic anion redistribution between [PF_6_]^−^ and [TFSI]^−^ [34], thereby weakening the cation‐anion interaction and facilitating the formation of a more homogeneous and stable ternary heterojunction.

To further demonstrate our hypothesis, we performed density functional theory (DFT) calculations combined with ab initio molecular dynamics (AIMD) simulations to investigate ion dynamics and evaluate the Gibbs free energy on a picosecond timescale (Figure 1c, Figure S1 and Video S1). The computational details of the AIMD simulations are provided in Note S1. As shown in Figure 1c and Figure S1, [PF_6_]^−^ preferentially resides near [EMIM]^+^ rather than remaining associated with [DMIM]^+^, indicating that the interaction between [PF_6_]^−^ and [EMIM]^+^ is thermodynamically favored. This preference originates from differences in charge distribution: [EMIM]^+^ retains a more localized charge around the imidazolium ring, while [DMIM]^+^ exhibits a more delocalized charge due to its long alkyl chains, resulting in a weaker electrostatic interaction with [PF_6_]^−^. Consequently, the incorporation of I_2_ promotes the redistribution of [PF_6_]^−^ toward the energetically favored [EMIM]+ environment, weakening the effective ion pairing in I_1_ and likely disrupting its alkyl‐chain packing.

In order to clarify the role of ion exchange, thin films of pristine P3HT, P3HT:I_1_, and P3HT:I_1_:I_2_ were investigated using Raman mapping and high‐angle annular dark‐field scanning transmission electron microscopy (HAADF‐STEM) (Figure 1d). The full Raman mapping procedure is detailed in Figure S2. The mapping analysis focused on the C═C and C─C vibration modes of P3HT to assess the distribution of P3HT in the film. The Raman mapping images revealed that the binary blend (blue regions) retained a rigid ionic matrix that remained largely intact even after ion‐gel washing, whereas the ternary blend (red regions) exhibited a more homogeneous Raman intensity without defects. HAADF‐STEM images also displayed pronounced phase separation in the P3HT:I_1_ film, while the P3HT:I_1_:I_2_ film exhibited a more homogeneous heterojunction between the CP and ionic liquids. Consistent morphological trends were further observed in atomic force microscopy (AFM) images of P3HT:I_1_ and P3HT:I_1_:I_2_ (Figure S3).

Since changes in thin‐film morphology and molecular ordering are often reflected in optical properties, both binary and ternary blends were analyzed in the solution and thin‐film states using UV‐Vis spectroscopy (Figure 1e). Upon binary blending, the P3HT:I_1_ film exhibited a more pronounced shoulder feature but also showed a blue shift of the 0–1 transition from 547 to 540 nm together with peak broadening, reflecting an unstable polymer‐ion environment. However, the ternary blend system completely recovered these I_1_‐induced spectral changes. This indicates that the structural disturbance of P3HT caused by I_1_ blending is effectively alleviated in the ternary blend film through the ion‐exchange process. We also conducted optical analysis of the three sample in the solution phase (Figure S4a). All chloroform solutions showed a blue‐shifted absorption maximum at 448 nm, while a pronounced shoulder peak at 600 nm, corresponding to the vibronic signature of P3HT aggregation, was observed only in the ternary system (P3HT:I_1_:I_2_) [35]. These observations suggest that the coexistence of I_1_ and I_2_ in solution alters the local polymer‐ion interaction environment of P3HT, thereby promoting pre‐aggregated chain conformations prior to film formation. In addition, the P3HT:I_2_ solution did not exhibit any change in the absorption peak relative to that of neat P3HT (Figure S4b). Finally, we attempted to fabricate a P3HT:I_2_ thin film to investigate its optical properties. However, as discussed above, a strongly de‐wetted morphology was observed due to pronounced surface energy mismatch (Figure S4c). Overall, the long alkyl chain of I_1_ enhances compatibility with the alkyl side chains of P3HT through favorable van der Waals interactions, mitigating the surface‐energy mismatch and supporting continuous film formation. Meanwhile, I_2_ induces thermodynamically favorable anion redistribution, in which [PF_6_]^−^ preferentially associates with [EMIM]^+^, weakening the original ionic organization of I_1_ and promoting a more homogeneous and stable polymer–ionic matrix.

### 2.2 Stable Electrochemical Doping in Ion‐Exchange‐Driven Ternary Organic Blend Devices

**FIGURE 2:**
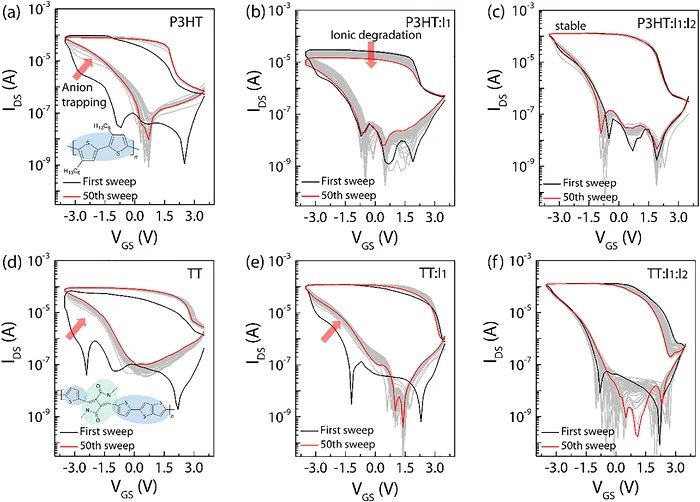
Universal electrochemical doping enhancement in ternary‐blend systems across homopolymer (P3HT) and donor‐acceptor polymer (TT) platforms. (a‐c) Cycling stability showing 50 consecutive transfer curves for (a) P3HT, (b) P3HT:I_1_, and (c) P3HT:I_1_:I_2_. The inset shows the molecular structure of P3HT, highlighting its π‐π conjugated backbone. (d‐f) Cycling stability showing 50 consecutive transfer curves for (d) TT, (e) TT:I_1_, and (f) TT:I_1_:I_2_. The inset shows the molecular structure of the TT polymer, highlighting its π‐π conjugated backbone. All transfer characteristics were measured by sweeping the gate voltage from 3.5 V to −3.5 V at a drain voltage (V_DS_) of −50 mV.

Before demonstrating the electrical performance of the ternary blend, we first examined the stabilization of [TFSI]^−^ and [PF_6_]^−^ anions by fabricating two‐terminal devices using three types of samples (P3HT, P3HT:I_1_, and P3HT:I_1_:I_2_) (Figure S5). Negligible off‐current was observed under various V_DS_ conditions for all samples, indicating that the films remained in a neutral state without electrochemical doping. The electrochemical stability and operational efficiency of the devices were further assessed through transfer characteristics measured over 50 consecutive cycles (Figure 2a–c). Over repeated cycling, the pristine P3HT device exhibited an increase in off‐current, attributed to P3HT oxidation induced by progressive anion trapping [36, 37]. In contrast, the P3HT:I_1_ binary device showed a gradual decrease in on‐current, indicating device degradation arising from unstable ionic interactions. Notably, the P3HT:I_1_:I_2_ ternary device demonstrated exceptional stability and consistent performance throughout the cycling test, suggesting that the ternary bulk heterojunction not only improves miscibility but also stabilizes electrochemical doping under gate bias.

In addition, transfer characterization was performed using DPP‐based TT polymer to evaluate the general applicability of the ion‐exchange strategy (Figure 2d–f). Given that DPP‐based TT polymers possess highly crystalline push‐pull backbones that typically limits ion‐gel miscibility, they provide a challenging platform for assessing the general applicability of this approach. However, as observed for the ternary blend of P3HT, the TT:I_1_:I_2_ device also displayed robust stability during repeated cycling tests. These consistent results in both thiophene‐ and DPP‐based polymers highlight that the ion‐exchange‐driven ternary blend strategy is broadly applicable across chemically and structurally diverse conjugated polymer systems. The key performance metrics, including the on/off ratio, memory window, and threshold voltage after 50 cycles, are summarized in Figures S6 and S7. Ternary blend devices based on both P3HT and TT polymers exhibited the highest on/off ratios, stable V_TH_ values, and consistent memory windows throughout cycling. To further demonstrate the stability and reliability of the ternary‐blend strategy, we evaluated the device yield by measuring the transfer characteristics of ten representative P3HT:I_1_:I_2_ devices. First, the coefficient of variation of the on/off ratio and the ranges of the threshold voltage (V_TH_) and hysteresis window (V_window_) were extracted from Figure 2a–c and used as reference criteria for stable and reliable device operation (Figure S8). The P3HT:I_1_:I_2_ device exhibited the lowest variation in these parameters, supporting the improved operational stability of the ternary blend system. Using these reference values, all ten devices satisfied the predefined criterion for the V_TH_ range (Figure S9c). However, D1 exceeded the criterion for the V_window_ range, whereas D8 exceeded the criterion for the CV of the on/off ratio (Figure S9b,d). Therefore, eight of the ten representative devices satisfied all stability criteria, corresponding to an 80% device yield across ten independently prepared batches. These improvements clearly demonstrate the advantage of the ternary blend strategy in IG‐OECTs.

### 2.3 Neuromorphic Functionality and Ion‐Mediated Retention Mechanism in Ternary Organic Blend Devices

**FIGURE 3:**
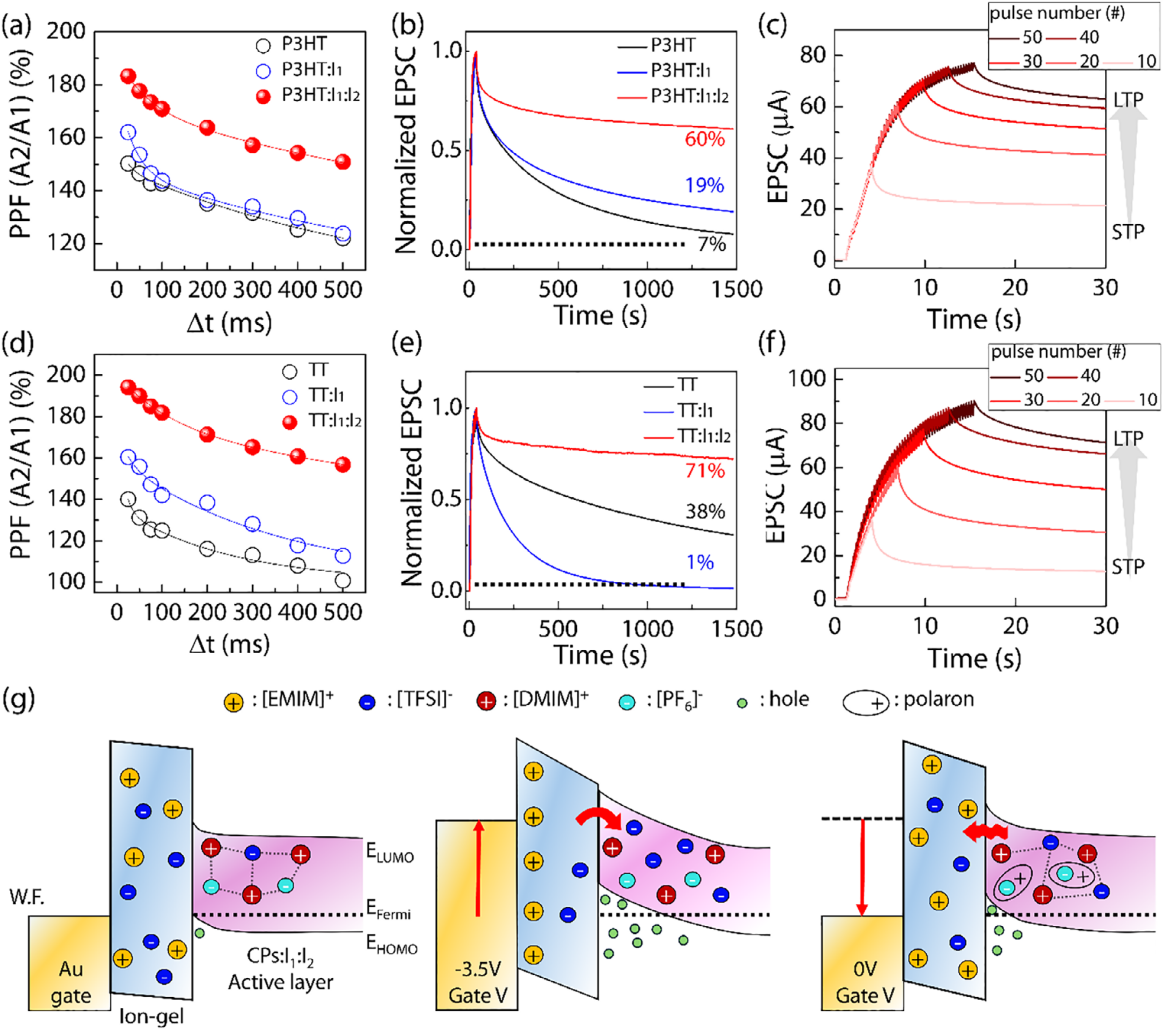
Universal neuromorphic behavior and retention mechanism in ternary‐blend organic electrochemical transistors. (a) Paired‐pulse facilitation (PPF) index of P3HT‐based devices (P3HT, P3HT:I_1_, and P3HT:I_1_:I_2_), defined as A_2_/A_1_, measured at time intervals of 25, 50, 75, 100, 200, 300, 400, and 500 ms using two consecutive pulses (−3.5 V, 100 ms). (b) Long‐term memory performance measured over 1500 s using 50 pulses (−3.5 V, 100 ms). (c) Spike‐number‐dependent plasticity (SNDP) measured with varying spike numbers (10, 20, 30, 40, and 50 pulses). (d‐f) Universality evaluation of the ternary blend system using TT‐based devices (TT, TT:I_1_, and TT:I_1_:I_2_). (d) PPF index, (e) long‐term memory, and (f) SNDP were measured under the same conditions as in (a‐c), respectively. (g) Schematic energy band diagrams illustrating the accumulation and retention mechanisms of the CPs:I_1_:I_2_ device. From left to right: the initial state before applying a negative gate bias, the accumulation state under bias, and the retention state after removing the gate bias.

The neuromorphic functionality of the devices was evaluated by emulating key neural processes, including short‐term and long‐term plasticity, through paired‐pulse facilitation (PPF) and long‐term potentiation (LTP) measurements (Figure 3a,b). The excitatory postsynaptic current (EPSC) amplitudes were quantified using the PPF index, defined as PPF = (A_2_/A_1_) × 100%, where A_1_ and A_2_ correspond to the EPSC amplitudes induced by the first and second stimuli, respectively. The P3HT:I_1_:I_2_ device demonstrated enhanced facilitation across all pulse intervals, achieving a maximum PPF value of 155% at Δt = 500 ms. As the PPF index exhibited an exponential decay with increasing pulse interval, the temporal dynamics were analyzed using the fitting equation [16, 22, 38, 39]:

(1) $$I_{PPF}=100+C_{1}\exp\left(-\frac{\Delta t}{\tau 1}\right)+C_{2}\exp\left(-\frac{\Delta t}{\tau 2}\right)$$

The longer time constant, τ_2_, which is associated with slow ion‐related processes (e.g., trapping and relaxation), was 1476 ms for the P3HT:I_1_:I_2_ device, which is substantially higher than those of pristine P3HT (362 ms) and P3HT:I_1_ (475 ms), as summarized in Table S1. For long‐term potentiation measurements, 50 gate pulses (an interval of 100 ms, a width of 100 ms, and VGS = −3.5 V) were applied to each device. The P3HT:I_1_:I_2_ device exhibited superior long‐term memory performance, retaining 60.7% of the potentiated EPSC after 1500 s, whereas pristine P3HT and P3HT:I_1_ retained only 7% and 19%, respectively. The device responses were further analyzed as a function of pulse number, amplitude, width, and frequency to emulate synaptic plasticity under various stimulation conditions (Figure 3c and Figure S10). Notably, pulse‐width‐dependent measurements confirmed effective EPSC modulation even under short gate pulses as short as 5 ms, indicating that the ternary device can respond to brief stimulation while maintaining gradual synaptic‐weight modulation. This behavior is attributed to counterion redistribution, which reduces [PF_6_]^−^ confinement within I1‐rich ionic domains and facilitates its migration toward the polymer backbone under brief stimulation.

To demonstrate the universality of the ternary blend system, we applied the same neuromorphic characterization to a DPP‐based TT polymer with a highly crystalline backbone. Under identical measurement conditions, the TT:I_1_:I_2_ devices exhibited enhanced STP behavior (PPF = 162% at Δt = 500 ms and τ_2_ = 1835 ms) and improved LTP retention (71.2% at 1500 s) compared with the TT and TT:I_1_ devices (Figure 3d,e and Table S2). Moreover, synaptic plasticity under various stimulation conditions was also observed in the TT:I_1_:I_2_ device (Figures 3f and S11). These results confirm that the ion‐exchange‐driven ternary strategy is broadly applicable across chemically distinct CP systems. A comparison with representative ion‐gel‐gated organic electrochemical transistors is provided in Table S3 [40, 41, 42, 43, 44, 45, 46, 47]. Although several previous studies reported slightly higher retention values, they generally required substantially higher operating voltages, longer pulse widths, or newly synthesized materials. In contrast, the proposed ternary system achieves a well‐balanced combination of long‐term retention, low‐voltage operation, and a simple blending strategy. Furthermore, extended long‐term memory (LTM) retention over 25 h and multi‐level storage capability with up to 50 distinguishable conductance states were demonstrated for the ternary blend device (Figures S12 and S13). Under these stimulation conditions, the P3HT:I_1_:I_2_ and TT:I_1_:I_2_ ternary blend devices exhibited gradual and stable modulation of synaptic weight without abrupt potentiation or depression, thereby enabling reliable LTM behavior and multi‐state data storage suitable for neuromorphic hardware applications.

Figure 3g schematically illustrates the operating principle and memory retention behavior of the CP:I_1_:I_2_ device using energy band diagrams. In the initial stage, a stabilized ionic state is formed by spontaneous ion exchange within the polymer matrix. In accumulation mode under a gate voltage of −3.5 V, this ionic state facilitates deep anion penetration into the semicrystalline domains of the CPs, accompanied by an expansion of the lamellar stacking distance. Upon removal of the gate bias, steric confinement and electrostatic attraction from the alkyl side chains of the polymer and bulky cations suppress the de‐doping process by effectively trapping the anions, resulting in enhanced long‐term memory retention.

### 2.4 In Situ Observation of Ion‐Induced Molecular Reorientation in Ternary Blend Organic Systems via GIWAX Measurement

**FIGURE 4:**
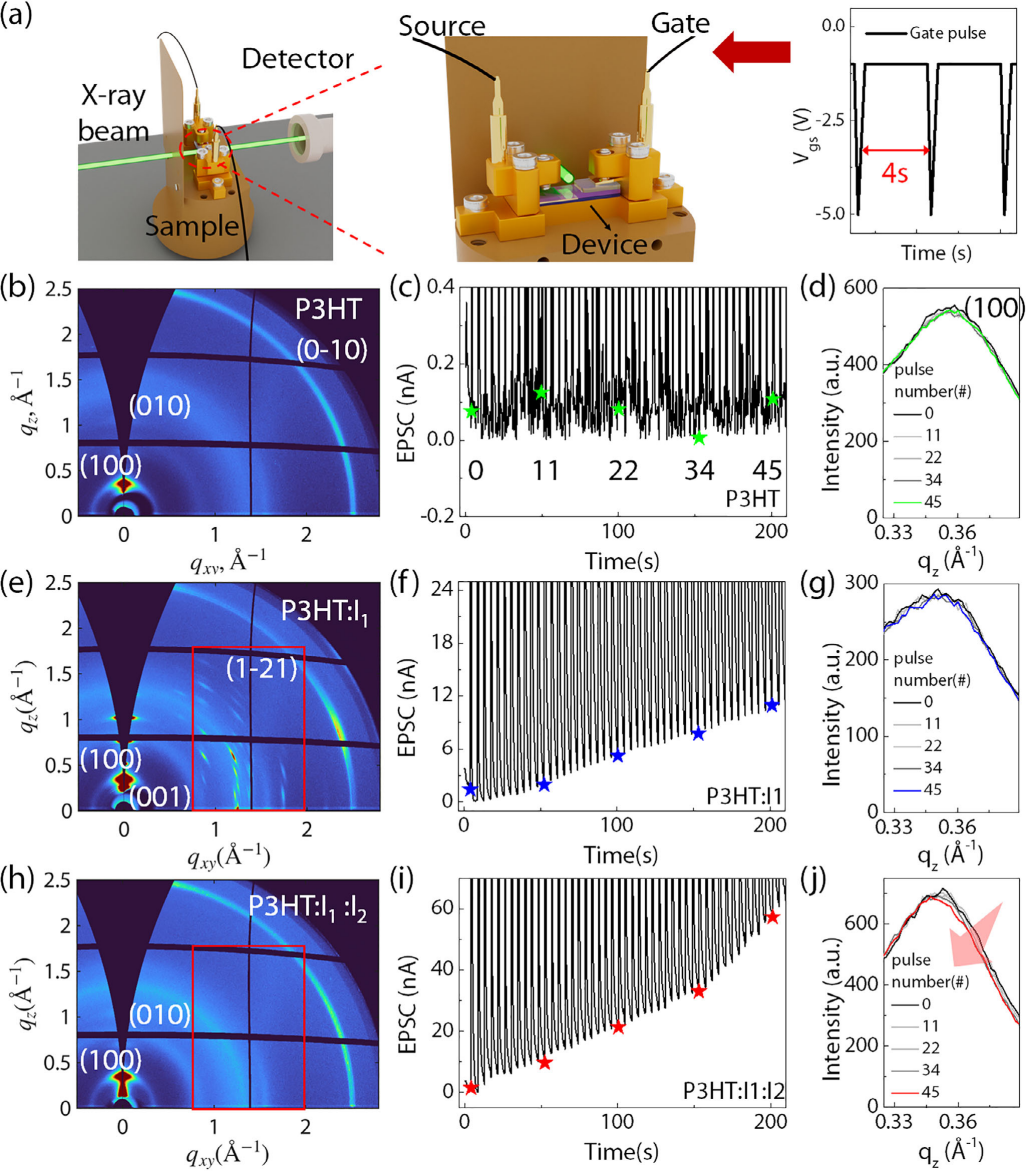
In situ GIWAXS analysis of ion‐induced structural evolution in ternary‐blend polymer systems. (a) Schematic illustration of the In situ GIWAXS measurement setup. A two‐terminal jig and device were configured for the In situ measurements. All measurements were performed using a gating pulse of −5 V (100 ms width) and a read pulse of −1 V (4000 ms width). (b), (e), and (h), 2D GIWAXS patterns of (b) P3HT, (e) P3HT:I_1_, and (h) P3HT:I_1_:I_2_. The (100) diffraction peak corresponds to the lamellar stacking of P3HT, while the (001)/(1‐21) peaks originate from the monoclinic structure of I_1_. (c), (f), and (i), Corresponding EPSC accumulation extracted for (c) P3HT, (f) P3HT:I_1_, and (i) P3HT:I_1_:I_2_ during the In situ measurements. The 2D GIWAXS images were acquired at the time points indicated by stars, corresponding to the 0th, 11th, 22nd, 34th, and 45th pulses. (d), (g), and (j), The corresponding 1D line‐cut profiles extracted along the out‐of‐plane direction for (d) P3HT, (g) P3HT:I_1_, and (j) P3HT:I_1_:I_2_, highlighting the (100) lamellar stacking peak.

This pronounced neuromorphic performance, particularly the strong long‐term retention behavior, is attributed to distinctive molecular‐level reorientation within the polymer matrix during the ionic‐gating process. To directly elucidate how the ternary bulk heterojunction modulates molecular orientation under operando conditions, In situ grazing‐incidence wide‐angle X‐ray scattering (GIWAXS) was performed on two‐terminal devices based on neat P3HT, P3HT:I_1_, and P3HT:I_1_:I_2_ films (Figure 4a). Figure 4a shows a schematic illustration of the In situ GIWAXS measurement setup (see the photographs in Figure S14). This device configuration includes a small gap between the Au electrode and the ion‐gel gate, enabling the channel to be directly exposed to the X‐ray beam while allowing controlled bulk electrochemical doping.

As shown in Figure 4b,e,h, the 2D GIWAXS patterns at the 0th pulse reveal the clear effect of the ternary blend system on the initial structures of pristine P3HT, P3HT:I_1_, and P3HT:I_1_:I_2_. All devices showed the conventional lamellar stacking peak of P3HT corresponding to the (100) plane, the halo peak of the ion‐gel corresponding to the (010) plane, and the Au electrode peak corresponding to the (0‐10) plane. The P3HT:I_1_ film exhibited strong diffraction peaks corresponding to the (001) and (1‐21) planes, related to the base‐centered monoclinic crystal structure of I_1_ (see Figures S15 and S16 for further details) [48, 49]. In contrast, although the P3HT:I_1_:I_2_ film contained the same amount of I_1_, the I_1_‐related diffraction peaks (highlighted by the red boxes) disappeared, suggesting that the addition of I_2_ weakens the original cation–anion interactions of I_1_ through ion exchange. This observation is consistent with the DFT/AIMD results shown in Figure 1c, where [PF_6_]^−^ preferentially associates with [EMIM]^+^, making the ion‐exchange process thermodynamically favorable. The resulting weakening of the original [DMIM]^+^[PF_6_]^−^ ion pairing provides a plausible explanation for the disappearance of the I_1_‐related crystalline diffraction peaks.

We plotted the EPSC responses during 50 consecutive pulses in Figure 4c,f,i. The P3HT device exhibited negligible EPSC accumulation during In situ measurements. Notably, despite the small gap between the electrode and the gel, both P3HT:I_1_ and P3HT:I_1_:I_2_ devices displayed clear EPSC accumulation caused by pulse‐driven bulk electrochemical doping. In particular, a higher EPSC level was observed in the ternary blend system. During the 50 consecutive pulses, a total of 100 2D GIWAXS images were collected for the three samples, and five representative images were selected for analysis and marked as stars in the EPSC plots. Each star indicates the pulse timing at which the corresponding GIWAXS image was acquired.

Further analysis of the out‐of‐plane 1D profiles under operando conditions revealed subtle but distinct changes in the lamellar stacking of P3HT:I_1_:I_2_, providing direct evidence of anion‐induced molecular reorientation. In the out‐of‐plane direction (Figure 4d,g,j and Figure S17), both pristine P3HT and P3HT:I_1_ films exhibited a characteristic diffraction peak corresponding to the (100) plane at *q_z_* = 0.359 Å^−^^1^ and *q_z_* = 0.355 Å^−^^1^ at both the 0th pulse and 45th pulse, respectively. In contrast, the P3HT:I_1_:I_2_ film showed a clear shift of the (100) peak from *q_z_* = 0.355 Å^−1^ to 0.345 Å^−1^ at the 45th pulse without noticeable full width at half maximum (FWHM) broadening. The extracted *q_z_* shifts, FWHM values, and correlation lengths are summarized in Figure S18 and Tables S4 and S5. These results suggest that the observed structural response is more plausibly associated with electrochemical doping by the relatively small and rigid [PF_6_]^−^ anion, which promotes lamellar expansion while largely preserving the initial lamellar order.

To further clarify the origin of the *q_z_* shift, we performed In situ GIWAXS measurements on the three films under retention conditions (Figure S19). The shifted *q_z_* values of P3HT:I_1_:I_2_ remained unchanged during the subsequent retention measurement without additional gate pulses, despite continuous X‐ray exposure. This indicates that the observed *q_z_* shift originated from pulse‐driven electrochemical doping rather than beam‐induced damage. These results from In situ GIWAXS demonstrate that the spontaneous ion‐exchange‐driven ternary bulk heterojunction enables efficient [PF_6_]^−^ penetration into polymer lamellar domains under 100 ms gate pulses and maintains the molecular reorientation induced by electrochemical doping under retention conditions, ultimately contributing to enhanced long‐term memory retention.

### 2.5 In Situ Raman and Impedance Analyses Revealing Ion‐Mediated Polaron Formation and Bulk Electrochemical Doping

**FIGURE 5:**
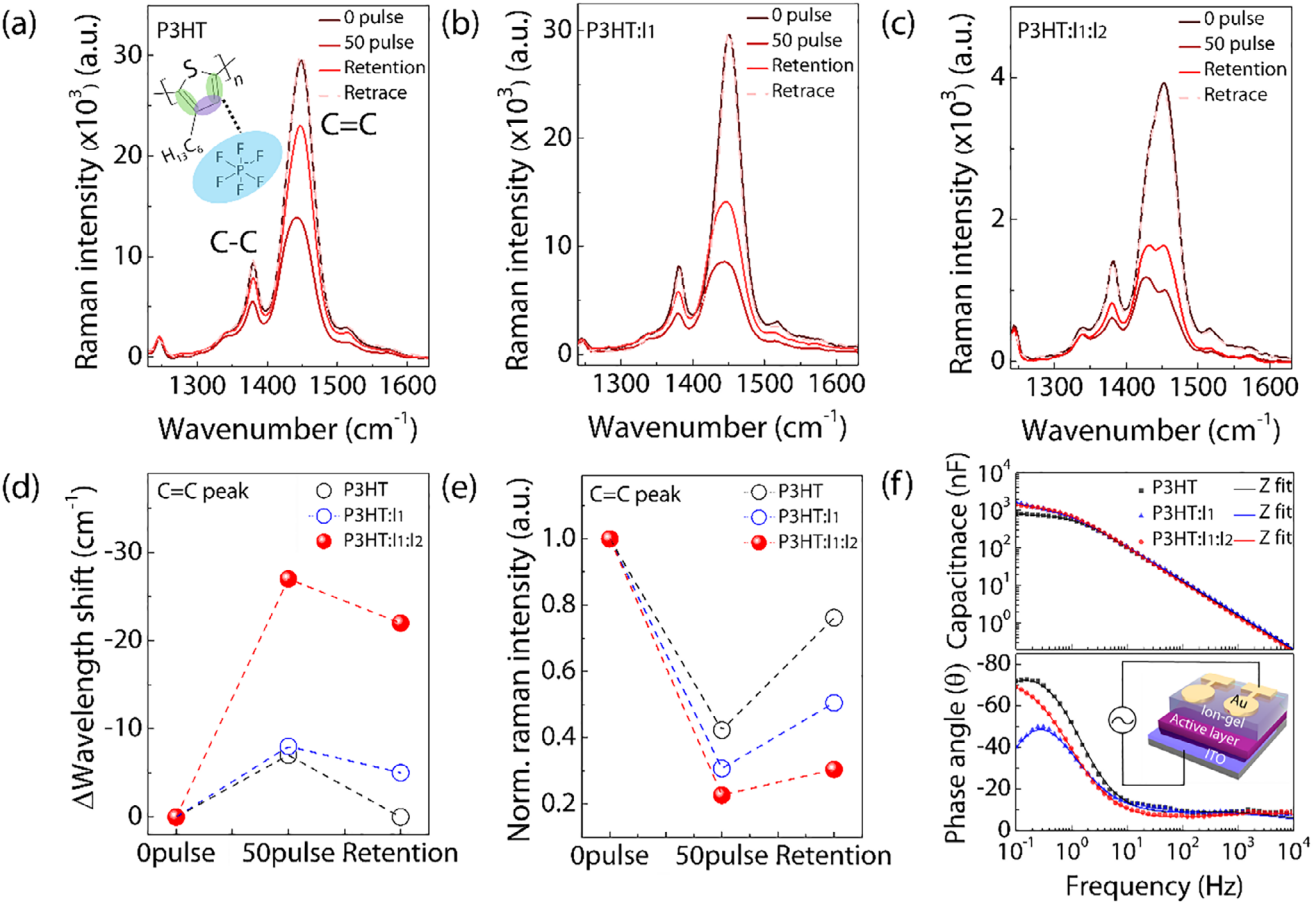
Direct spectroscopic and impedance evidence of ion‐polaron interactions governing electrochemical doping in ternary‐blend systems. (a‐c) Evolution of Raman intensity corresponding to the P3HT polaron peaks (C═C and C─C modes) during In situ electrochemical doping for (a) P3HT, (b) P3HT:I_1_, and (c) P3HT:I_1_:I_2_. The inset illustrates the C═C and C─C bonds of P3HT and the interaction between the π‐π conjugated backbone and [PF_6_]^−^ anions associated with polaron formation. (d, e) Comparison of (d) peak position (wavelength shift) and (e) normalized Raman intensity extracted from the In situ tracking in (a‐c). (f) Phase angle and capacitance as a function of frequency for the three devices, based on a metal‐insulator‐semiconductor (MIS) structure. The inset shows the MIS configuration adapted from a previous study.

To directly probe whether the structural changes observed in the ternary blend system via In situ GIWAXS facilitate the electrochemical doping of P3HT into a polaronic state during ion gating, In situ Raman spectroscopy measurements were performed (Figure 5a–c). Each sample was subjected to 50 potentiation pulses (−3 V, 100 ms), and the characteristic Raman modes of P3HT, including the C─C stretching mode at ∼1380 cm^−^^1^ and the C═C stretching mode at ∼1451 cm^−^^1^ (shown in the inset), were monitored under the pristine state (0 pulses), doped state (50 pulses), retention state (after 200 s), and retrace state. After the application of 50 pulses, all three samples exhibited a decrease in Raman intensity accompanied by a redshift of the C═C stretching mode, consistent with polaron formation associated with bond softening and elongation [50, 51]. Notably, the P3HT:I_1_:I_2_ ternary film exhibited the most pronounced spectral evolution, characterized by the largest redshift and the slowest recovery of Raman intensity during the retention period (Figure 5d,e). Under identical pulse conditions (amplitude, width, and frequency), the P3HT:I_1_:I_2_ ternary device exhibited a redshift of 27 cm^−^^1^, which is substantially larger than those observed for pristine P3HT (∼ 7 cm^−^^1^) and P3HT:I_1_ (∼ 9 cm^−^^1^). To further evaluate the de‐doping kinetics of the three samples, the recovery of Raman intensity was monitored over a 200 s retention period. The quantitative analysis of normalized intensity recovery revealed that the P3HT:I_1_:I_2_ ternary device exhibited the lowest recovery value (0.08), whereas P3HT and P3HT:I_1_ showed higher recovery values of 0.34 and 0.20, respectively.

To clarify the ion‐injection dynamics and charge‐storage mechanisms in the three films, we conducted electrochemical impedance spectroscopy (EIS) measurements using metal‐insulator‐semiconductor (MIS) devices (Figure 5f) [52]. The capacitance‐frequency (*C‐f*) characteristics showed that both the P3HT:I_1_ and P3HT:I_1_:I_2_ devices exhibit high capacitance values of approximately 1400 nF at 100 mHz, which were significantly higher than that of pristine P3HT (800 nF). To further investigate the origin of the enhanced capacitance, the phase‐angle characteristics presented in the lower panels of Figure 5f were analyzed. The P3HT:I_1_:I_2_ ternary device exhibited a phase angle approaching ‐75° at 100 mHz, while the P3HT:I_1_ device showed a considerably smaller value of ‐40°. The larger magnitude of the negative phase angle indicates a more capacitive response consistent with volumetric ionic charging, suggesting that ions in the P3HT:I_1_:I_2_ system are not confined to the ion‐gel‐gate/CP interface but can effectively penetrate the bulk of the active layer [53, 54]. To eliminate apparent capacitance contributions arising from resistive components, the intrinsic capacitance values were extracted through impedance fitting based on an equivalent circuit model of the Nyquist plots (Figure S20 and Table S6). The extracted intrinsic capacitance of the P3HT:I_1_:I_2_ device reached 1400 nF, which is higher than that of the P3HT:I_1_ device (1000 nF) despite their similar apparent capacitance values (∼1400 nF) in the *C‐f* plots. This increase further supports the superior bulk charge‐storage capability of the ternary blend system. Therefore, the In situ spectroscopic and electrochemical analyses clearly demonstrate that the enhanced neuromorphic performance and structural modulation of the ternary blend system originate from spontaneous ion‐exchange‐driven [PF_6_]^−^ penetration and electrochemical doping within the bulk polymer matrix.

### 2.6 Hardware‐Aware Neural Network Performance Enabled by Ternary Blend Organic Systems

**FIGURE 6:**
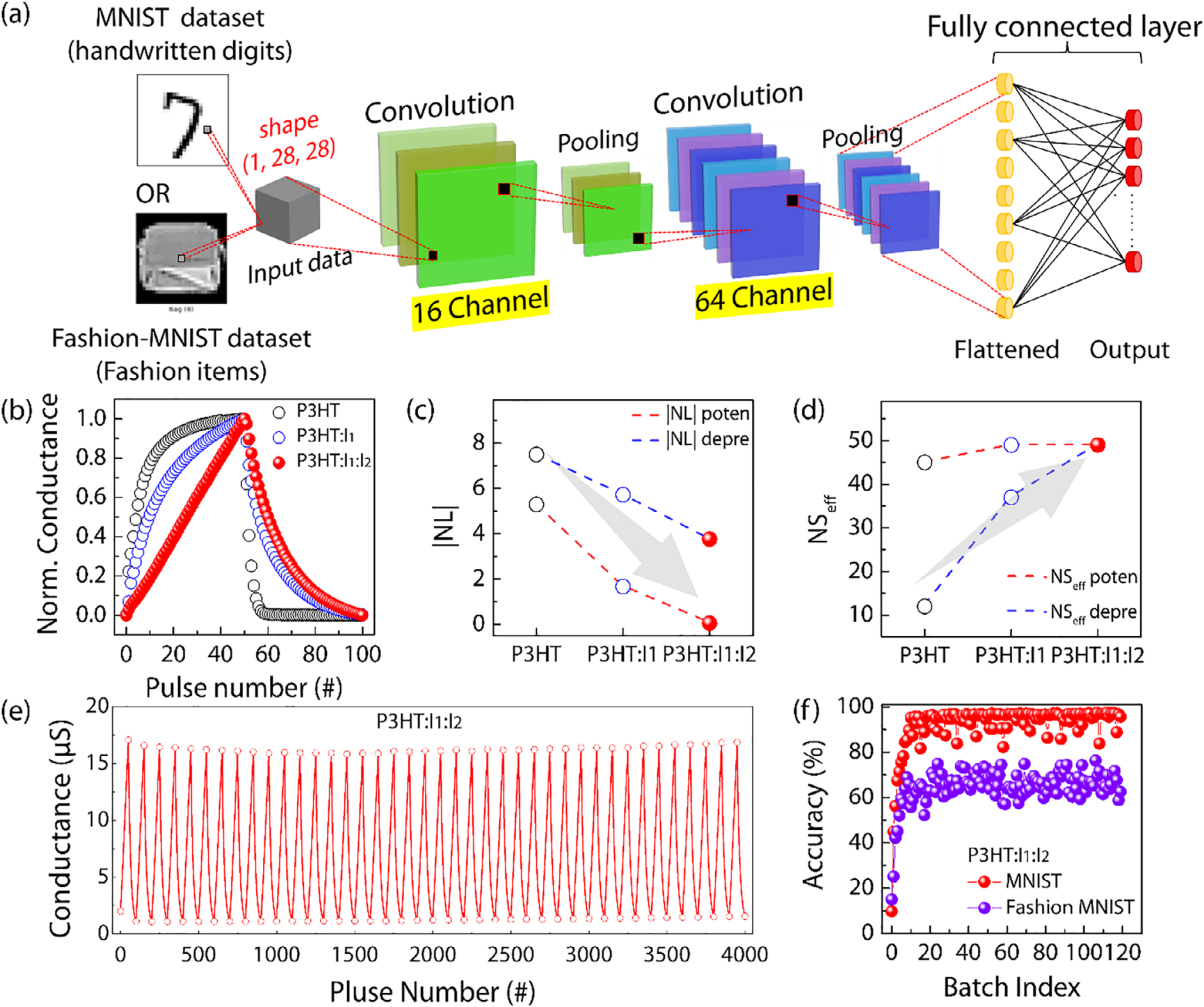
Hardware‐aware neural network validation of ternary‐blend synaptic devices using MNIST classification. (a) Schematic of a hardware‐aware convolutional neural network (CNN) employing bulk‐heterojunction FET synapses for ex situ training. (b) Normalized conductance change during long‐term potentiation (LTP) and long‐term depression (LTD) over 50 pulses (−3.5 V, 100 ms for LTP and +3.5 V, 100 ms for LTD). (c, d) Comparison of (c) nonlinearity and (d) effective number of conductance states extracted from the LTP/LTD characteristics in (b). (e) Endurance characteristics over 40 cycles of LTP/LTD, demonstrating stable and reliable operation. All gate pulses were applied at ±3.5 V with a pulse width of 100 ms. (f) Classification accuracy of the hardware‐aware CNN employing the proposed synaptic devices, evaluated on the MNIST and Fashion‐MNIST datasets.

The proposed spontaneous ion‐exchange‐driven bulk heterojunction IG‐OECT device was employed as a synaptic element to represent trainable weights for the *ex situ* training of a hardware‐aware convolutional neural network (CNN). In the CNN architecture designed for image classification, input images, such as MNIST handwritten digits or grayscale images with dimensions of 1 × 28 × 28 are first processed through a series of convolutional layers (Figure 6a) [55, 56]. Figure 6a illustrates the workflow of the CNN for image classification: the input image is progressively transformed through convolutional and pooling layers, where spatial resolution decreases while channel depth increases, yielding a compact yet information‐rich representation. The resulting feature maps are then flattened and passed through fully connected layers, which utilize the extracted hierarchical features to perform classification.

To evaluate the synaptic characteristics of the devices in response to CNN weight updates, the normalized long‐term potentiation and depression (LTP and LTD), which represent the write and erase characteristics of the device, were measured for the three samples with 50 pulses of potentiation (−3.5 V, 100 ms) and 50 pulses of depression (+3.5 V, 100 ms) (Figure 6b–d). Among the three samples, the P3HT:I_1_:I_2_ ternary device exhibited the most symmetric conductance modulation during both the 50 pulses of potentiation and depression. We calculated the non‐linearity (|NL|) and the effective number of states (NS_eff_), defined as the number of resolvable conductance states under a conductance variation of at least 0.1% between two conductance levels in potentiation and depression, to quantify the synaptic weight update characteristics. The |NL| fitting curves are shown in Figure S21. The |NL| values for both potentiation and depression decreased from the P3HT to P3HT:I_1_:I_2_ (achieving |NL|_poten_ = 0.001 and |NL|_depre_ = 3.76 in P3HT:I_1_:I_2_), and the NS_eff_ values increased from P3HT to P3HT:I_1_:I_2_, ultimately achieving 49 effective states for both potentiation and depression.

The cycling stability of the P3HT:I_1_:I_2_ ternary device was further evaluated to ensure reliable synaptic operation during CNN training (Figure 6e). The device was subjected to 4000 consecutive potentiation pulses under a gate voltage of −3.5 V with a pulse width of 100 ms. Key performance parameters were summarized at intervals of 1000 pulses (Figure S22). In addition, pulse sequences of 256 pulses (= 2^8^ pulses) were used to assess the 8‐bit operation capability (Figure S23) [14, 20, 57]. Stable operation over 40 cycles of 100 pulses and 15 cycles of 256 pulses confirmed the robustness and endurance of the devices. The hardware‐aware CNN model incorporating the proposed synaptic characteristics was evaluated using the MNIST dataset, consisting of 100 000 training samples and 30,000 test samples with a resolution of 28 × 28. The computational details are provided in Note S2) [58, 59, 60, 61, 62, 63]. Under a device‐aware training protocol that accounts for the 50‐level potentiation/depression nonlinearity and weight‐quantization constraints, the network achieved test accuracies of 97.89% on MNIST and 76.15% on Fashion‐MNIST (Figure 6f). These results demonstrate that the proposed synaptic device and training approach maintain high classification accuracy despite intrinsic device nonidealities, supporting their potential application for scalable neuromorphic CNN accelerators.

## 3 Conclusion

In conclusion, we demonstrate a ternary blend strategy for organic neuromorphic devices that enables stable and efficient electrochemical doping through ion‐exchange‐mediated polymer‐ion interactions. The incorporation of two ionic liquids induces spontaneous anion redistribution, which improves the stabilization of relatively small anions (i.e., [PF_6_]^−^) within the conjugated polymer matrix. This ion‐exchange‐driven environment facilitates efficient electrochemical doping and leads to consistent device operation over repeated cycling, while enhancing both short‐ and long‐term synaptic plasticity. Notably, this behavior is observed across chemically distinct polymer systems, including the thiophene‐based P3HT and DPP‐based donor‐acceptor polymer (TT), indicating the universality of the approach. Under short gate pulses (100 ms), the ternary system enables efficient ion penetration into the polymer bulk, resulting in lamellar expansion and persistent polaron formation, as directly evidenced by In situ GIWAXS and Raman spectroscopy measurements, respectively.

The stabilized doping dynamics further yield highly linear and symmetric weight updates, which are essential for reliable neuromorphic computing. When implemented in a hardware‐aware convolutional neural network, the devices achieve high classification accuracies on both MNIST (97.89%) and Fashion‐MNIST datasets (76.15%), demonstrating their practical applicability despite intrinsic device non‐idealities. Furthermore, the proposed strategy is well suited to scalable manufacturing through solution‐based printing techniques, such as inkjet and roll‐to‐roll printing, offering a practical route toward large‐area, high‐density neuromorphic arrays. These findings establish the ion‐exchange‐driven ternary blend as an effective strategy for scalable, low‐temperature, and mechanically flexible neuromorphic electronics with system‐level functionality.

## 4 Experiment Section

### 4.1 Materials

Poly(3‐hexylthiophene) (P3HT) and DP‐PDPP2T‐TT (TT) were purchased from 1‐Material Inc. (Quebec, Canada) and used as received. The poly (vinylidene fluoride‐co‐hexafluoropropylene) (PVDF‐HFP) (Mw = 400000) was purchased from Sigma– Aldrich. 1‐dodecyl‐3‐methylimidazolium hexafluorophosphate ([DMIM]^+^[PF_6_]^−^, I_1_) was prepared according to procedures described in the literature. The 1‐Ethyl‐3‐methylimidazolium bis(trifluoromethylsulfonyl)imide ([EMIM]^+^[TFSI]^−^, I_2_) was purchased from TCI.

### 4.2 Device Fabrication

The ion‐gel solution was prepared with a weight ratio of PVDF‐HFP: I_2_: acetone = 1:4:7. The conjugated polymer was dissolved in chloroform at 3 mg mL^−1^ and stirred at 80 °C for several hours. To prepare for the CPs:I_1_ solution, I_1_ (7.57 mM) was added to the CP solution. For the CPs:I_1_:I_2_ solution, I_1_ (7.57 mM) and I_2_ (5.46 mM) were added to the CP solution. All solutions were filtered through a 0.45 µm filter and stirred at 80 °C overnight. Bottom‐contact substrates for IG‐OECTs were fabricated on glass or SiO_2_ wafers using Au/Ni (35 nm/5 nm) source and drain electrodes with a channel length of 50 µm and a width of 1000 µm. The cleaned substrates were sonicated sequentially in acetone and isopropyl alcohol for 10 min each and then dried at 60 °C for 20 min. After the cleaning process, the cleaned substrate was exposed to UV/Ozone treatment for 15 min to induce the modified surface. Before spin‐coating process, the solutions were heated to 80 °C for 5 min to resolve the spontaneous polymer aggregation and subsequently cooled for 5 min in N2‐filled glove box. The three types of solutions were spin‐coated onto the glass or SiO_2_ substrates as the active layer for 40 s at 1500 rpm. Finally, the pre‐heated ion‐gel solution was drop‐cast onto the active channel layer to fabricate the ion‐gel‐gate OECT, and then the devices were stored in a vacuum chamber for 1 h to evaporate the solvent.

### 4.3 Optical Properties Characterization

UV‐vis absorption spectra of the thin films were recorded in transmission mode by utilizing a Jasco UV‐770 spectrophotometer. The transmittance values (T) were converted to absorbance (A) according to the Beer‐Lambert law: A = −log10(T/T_0_), where T_0_ denotes the transmittance of a freshly cleaned quartz substrate. The solutions of three samples for the absorption spectra were contained in a 1.4×1.4×2 cm glass cuvette at a concentration of 0.03 mg mL^−1^ owing to the dynamic range of the spectrophotometer.

### 4.4 Morphological Characterization

High‐angle annular dark field‐scanning transmission electron microscopy (HAADF‐STEM) images were collected using a Tecnai G2 F30 S‐Twin microscope operated at 300 keV. The sample films for the HAADF‐STEM measurements were obtained by peeling the pre‐deposited films from a substrate and transferring them onto 200‐mesh copper grids (Electron Microscopy Sciences).

### 4.5 Structural Characterization

GIWAXS measurements were performed at the 3C‐SAXSl beam line in the Pohang Accelerator Laboratory (PAL). XPS data were obtained using a Veresprobe II (ULVAC‐PHI) instrument. ESR measurements were performed on a Bruker EMXplus X‐band (∼9.8 GHz) spectrometer at 25°C [64].

### 4.6 Electrical Properties Characterization

The electrical and neuromorphic performances were characterized using a Keithley 4200. An Au probe tip was used to apply gate pulses to the ion‐gel gate. Transfer curves (I‐V dual sweeps) were measured by sweeping V_GS_ from 3.5V to −3.5 V with a V_DS_ of ‐50 mV. To evaluate all synaptic properties, voltage pulses (V_GS_ = −3.5 V with a duration and interval of 100 ms) were applied at a V_DS_ = 50 mV. The on/off ratio was determined based on the *I*_DS_ at the V_GS_ = 0V and threshold voltage (V_TH_) was estimated as the gate voltage corresponding to an *I*_DS_ of 10^−5^A.

### 4.7 Evaluation of Non‐Linearity |NL|

For a precise analysis of the symmetric curve, we evaluated the non‐linearity (|NL|) using the following equation:

(2) $$G_{LTP}=B\;\left(1-e^{\left(-\frac{P}{A}\right)}\right)+G_{min}$$

(3) $$G_{LTD}=-B\;\left(1-e^{\left(-\frac{P-P_{max}}{A}\right)}\right)G_{max}$$

(4) $$B=\left(G_{max}-G_{min}\right)/\left(1e^{-\frac{P_{max}}{A}}\right)$$

where G_LTP_ and G_LTD_ represent the conductance of potentiation and depression, respectively. P denotes the number of states, and A is a factor that affects the nonlinear curve and is used to determine the NL value by fitting the LTP/D curve.

### 4.8 Raman Spectroscopy Characterization

Raman spectra were obtained in backscattering geometry by means of a WEVE MantaRay Renewal multi‐confocal Raman microscope. For spectral calibration, a silicon reference was employed under short exposure conditions (0.1 s).

### 4.9 Electrochemical Impedance Spectroscopy Characterization

P3HT, P3HT:I_1_, and P3HT:I_1_:I_2_ solutions were deposited on indium tin oxide (ITO) substrates that had been chemically cleaned using standard procedures. Subsequently, the pre‐heated ion‐gel solution was deposited on thin films to make the MIS configuration. These coated substrates were then transferred to a vacuum deposition chamber with the back side oriented upward, and a thin gold layer was thermally evaporated onto the film surface. Capacitance‐frequency (C‐f) characteristics were measured using a SP‐150e potentiostat in the frequency range from 10 MHz to 100 mHz at room temperature.

## Author Contributions

**Sungmin Lee**: conceptualization, methodology, software, data curation, investigation, validation, writing – original draft, writing – review and editing. **Hyeonryul Lee**: software, data curation, investigation, validation, methodology. **Liqiang Li**: methodology, software, data curation, investigation, validation, formal analysis. **Young‐Yong Kim**: methodology, data curation, investigation, validation, software. **Changhoon Lee**: methodology, software, data curation, investigation, validation. **Vivek Pratap Singh**: software, data curation, investigation, validation. **Zhongwu Wang**: methodology, software, data curation, investigation, validation. **Yong‐Ryun Jo**: methodology, software, data curation, investigation, validation. **Namsoo Lim**: methodology, software, data curation, investigation, validation. **Chandran Balamurugan**: methodology, software, data curation, investigation, validation, formal analysis. **Dongyeop Yang**: methodology, software, data curation, investigation, validation. **Sooncheol Kwon**: methodology, conceptualization, software, data curation, investigation, validation, formal analysis, supervision, funding acquisition, visualization, project administration, resources, writing – original draft, writing – review and editing. **Guanjie Wang**: methodology, software, data curation, investigation, validation. **Junyeong Sung**: methodology, software, data curation, investigation, validation. **Ji Hoon Shim**: methodology, software, data curation, investigation, validation.

## Conflicts of Interest

The authors declare no conflicts of interest.

## Supporting information




**Supporting File 1**: advs77486‐sup‐0001‐SuppMat.docx.

**Supporting File 2**: advs77486‐sup‐0002‐S1.mp4.

## Data Availability

The data that support the findings of this study are available from the corresponding author upon reasonable request.
