# Supplementary material for: Ion‐Exchange‐Driven Ternary Organic Neuromorphic Devices with High Efficiency and Stability
Source: Adv Sci (Weinh). 2026 Sep 8:e77486. Online ahead of print. doi: 10.1002/advs.77486 (PMC13554501; doi:10.1002/advs.77486)
Supplement: Supplementary file 1 [file ADVS-9999-e77486-s002.docx]

**Supporting Information**

**Ion-exchange-driven ternary organic neuromorphic devices with high efficiency and stability**

*Sungmin Lee, Vivek Pratap Singh, Hyeonryul Lee, Young-Yong Kim, Yong-Ryun Jo, Changhoon Lee, Ji Hoon Shim, Namsoo Lim, Guanjie Wang, Junyeong Sung, Dongyeop Yang, Zhongwu Wang, Liqiang Li* *, Chandran Balamurugan, and Sooncheol Kwon*^*^

S. Lee, G. Wang, J. Sung, D. Yang, Dr. C. Balamurugan, Prof. S. Kwon

Department of Energy and Materials Engineering, Dongguk University-Seoul, Seoul, 04620 Republic of Korea

Email: kwansc12@dongguk.edu

Dr. V. Singh, H. Lee

Department of Advanced Battery Convergence Engineering, Dongguk University-Seoul,

Seoul, 04620 Republic of Korea

Dr. V. Singh

Department of Electronics and Communication Engineering, Maulana Azad National Institute of Technology (MANIT), Bhopal, 462003, Madhya Pradesh, India

Dr. Y. Kim

Pohang Accelerator Laboratory, Pohang University of Science and Technology, Pohang 37673, Republic of Korea

Dr. Y. Jo

GIST Advanced Institute of Instrumental Analysis (GAIA), Electron Microscopy Laboratory,

Gwangju Institute of Science and Technology, Gwangju 61005, Republic of Korea

Dr. C. Lee

Max Planck POSTECH Center for Complex Phase of Materials, Pohang University of

Science and Technology, Pohang 37673, Republic of Korea

Dr. J. Shim

Department of Chemistry, Pohang University of Science and Technology, 77 Cheongam-Ro,

Nam-gu, Pohang 37673, Republic of Korea

Dr. N. Lim

Research Institute of Molecular Alchemy, Gyeongsang National University, 501 Jinju-daero,

Jinju, South Gyeongsang, 52828, Republic of Korea

Prof. Z. Wang, Prof. L. Li

State Key Laboratory of Advanced Materials for Intelligent Sensing & Key Laboratory of

Organic Integrated Circuit, Ministry of Education & Tianjin Key Laboratory of Molecular

Optoelectronic Sciences, Department of Chemistry, Institute of Molecular Aggregation

Science, Tianjin University, Tianjin 300072, China

Keywords: organic neuromorphic device, ion exchange, ternary blend system, electrochemical doping, *in situ* observation

**Table S1.** Extracted parameters from PPF analysis of P3HT-based systems based on bi-exponential fitting.

Fitting parameters of the paired-pulse facilitation (PPF) characteristics for P3HT-based systems, obtained using a bi-exponential decay model. The parameters (C₁, C₂, τ₁, and τ₂) describe fast and slow ion relaxation dynamics associated with synaptic responses.

| **Device** | **C_1_** | **τ_1_ (ms)** | **C_2_** | **τ_2_ (ms)** |
| --- | --- | --- | --- | --- |
| P3HT | 12 | 49 | 49 | 624 |
| P3HT:I_1_ | 32 | 34 | 47 | 779 |
| P3HT:I_1_:I_2_ | 19 | 66 | 71 | 1475 |

**Table S2.** Extracted parameters from PPF analysis of TT-based systems based on bi-exponential fitting.

Fitting parameters of the paired-pulse facilitation (PPF) characteristics for TT-based systems, obtained using a bi-exponential decay model. The parameters (C₁, C₂, τ₁, and τ₂) represent fast and slow ion relaxation dynamics associated with synaptic responses.

| **Device** | **C_1_** | **τ_1_ (ms)** | **C_2_** | **τ_2_ (ms)** |
| --- | --- | --- | --- | --- |
| TT | 48 | 13 | 36 | 244 |
| TT:I_1_ | 15 | 23 | 59 | 362 |
| TT:I_1_:I_2_ | 26 | 122 | 74 | 1835 |

**Table. S3** Comparison of long-term retention performance and operating conditions of representative ion-gel-gated organic electrochemical transistors reported in the literature.

| Materials | Gate bias | Pulse width/interval | pulse # | Retained EPSC% | Ref. |
| --- | --- | --- | --- | --- | --- |
| P3HT/[DMIM][PF_6_][EMIM][TFSI]  DPP2T-TT/[DMIM][PF_6_][EMIM][TFSI] | -3.5V | 0.1s/0.1s | 50 | 60% at 1500s | This work |
|  | -3.5V | 0.1s/0.1s | 50 | 71% at 1500s |  |
| aDPP/gDpp | -3.5v | 0.1s/0.1s | 50 | >20% at 200s | [22] |
| P3HT/DTBP + Dual ion gel | -1V | 0.5s/1s | 100 | 34% at 1800s | [30] |
| PIBET-AO | -1.5V | 0.05s/0.05s | 200 | 81% at 1000s | [40] |
| P3HT/[BMIM][TFSI] film | -1.5V | 1s/1s | 100 | 34% at 240s | [41] |
| P3HT+Anion-Excessive ion gel | -1.5V | 0.18s/0.18s | 100 | 21.6% at 600s | [42] |
| PDPP3T + [EMIM][6M-TFSI] ion gel | -2.5V | 0.06s/0.06s | 10 | 34% at 150s | [43] |
| DPP-29SVS | -3V | 0.08s/0.08s | 50 | 1% at 800s | [44] |
| PEDOT:PSS + Ferroelectric gel | -3V | 0.1s/0.1s | 70 | ≈60% at 100s | [45] |
| PDPP3T + [EMIM][FSI] ion gel | -3V | 0.02s/0.02s | 10 | ≈50% at 100s | [46] |
| P(g2T-TT)/cross linker | -3V | 0.1s/0.1s | 300 | 64% at 1000s | [47] |

**Table S4.** Comparison of the initial and post-*in situ* *q_z_* values of the polymer lamellar (100) diffraction peaks for pristine P3HT, P3HT:I_1_, P3HT:I_1_:I_2_

| **Device** | **Initial q_z_ (Å⁻¹)** | **Post-*in situ* q_z_ (Å⁻¹)** |
| --- | --- | --- |
| P3HT | 0.359 | 0.355 |
| P3HT:I_1_ | 0.359 | 0.355 |
| P3HT:I_1_:I_2_ | 0.355 | 0.345 |

**Table S5.** FWHM and correlation length extracted from (100) diffraction peaks.

Full width at half maximum (FWHM) and correlation length values of the (100) lamellar diffraction peaks for each sample, obtained from Gaussian fitting of the profiles shown in Figure. 16. The correlation length was calculated using the Scherrer equation:
ξ = 2πK / Δq, where K is the shape factor (K = 1) and Δq is the FWHM.

| **Device** | **FWHM(Å^-1^)** | **Correlation length (Å)** |
| --- | --- | --- |
| P3HT | 0.090 | 69 |
| P3HT:I_1_ | 0.095 | 66 |
| P3HT:I_1_:I_2_ | 0.078 | 80 |

**Table S6.** Summary of extracted impedance fitting parameters and capacitance values from equivalent circuit analysis.

Impedance fitting parameters obtained from Nyquist plots using an equivalent circuit model. The extracted values were used to evaluate the intrinsic capacitance of each device.

| Device | R1 (Ω) | Q2 (F·s^a-1^) | R2 (Ω) | Q3 (F·s^a-1^) | R3 (Ω) |
| --- | --- | --- | --- | --- | --- |
| P3HT | 67749 | 0.496x10^-6^ | 97724 | 0.784x10^-6^ | 12.07x10^6^ |
| P3HT:I_1_ | 63815 | 1.312x10^-6^ | 1.520x10^6^ | 1.003x10^-6^ | 1.148x10^6^ |
| P3HT:I_1_:I_2_ | 61276 | 0.337x10^-6^ | 88596 | 1.453x10^-6^ | 17.58x10^6^ |

 **Figure S1.** Theoretical investigation of ion-exchange dynamics and cation-anion interactions.

**(a**, **b)** Chemical structures of [EMIM]⁺ and [DMIM]⁺ along with their corresponding charge distributions, highlighting differences in charge localization between the two cations. **(c)** Ab initio molecular dynamics (AIMD) simulation of the ion-exchange process on the picosecond timescale. [EMIM]⁺ and [DMIM]⁺ were used as cations, and [PF₆]⁻ as the anion, illustrating dynamic anion redistribution between the two cation environments.

 **Figure S2.** Raman mapping procedure and spatial distribution of P3HT before and after ion-gel washing. Schematic illustration of the Raman mapping process and corresponding spatial maps of P3HT:I_1_ and P3HT:I_1_:I_2_ films, obtained from the C=C (~1451 cm⁻¹) and C–C (~1380 cm⁻¹) stretching modes. The mapping was performed before and after ion-gel washing to evaluate the structural stability and polymer distribution. After deposition of the ion-gel solution, the samples were dried in an N₂-filled glovebox to remove residual solvent.

**Figure S3.** AFM characterization of morphology in binary and ternary blend systems. **(a, b)** Two-dimensional (2D) and three-dimensional (3D) tapping-mode AFM height images of P3HT:I_1_ and P3HT:I_1_:I_2_ films. All films were prepared under identical fabrication conditions to enable direct comparison of morphological features.

**Figure S4.** UV-Vis spectroscopy of polymer-ion interactions in solution and thin-film condition. **(a)** Normalized UV-Vis absorption spectra of P3HT, P3HT:I_1_, and P3HT:I_1_:I2 in chloroform solution (0.03 mg mL⁻¹). The inset shows time-dependent color evolution of the P3HT solution over 1 h, along with photographs of the corresponding thin films. **(b)** Normalized UV-Vis absorption spectra comparing P3HT:I_1_:I2 (red) and P3HT:I2 (green, P3HT:[EMIM]⁺[TFSI]⁻) to verify the role of ionic-liquid composition. **(c)** Optical image of the P3HT:I2 thin film. Severe dewetting is observed due to surface energy mismatch, where the ionic liquid ([DMIM]⁺[TFSI]⁻) forms isolated regions owing to its negligible vapor pressure.

**Figure S5.** Two-terminal electrical characteristics of binary and ternary blend systems. **(a)** Schematic illustration of the two-terminal device architecture. Au source and drain electrodes (channel length (*L*) = 20 μm, width (*W*) = 1 mm) were fabricated on the substrate. **(b, c)** Output characteristics of (b) P3HT (thiophene-based polymer) and (c) TT (DPP-based donor–acceptor polymer) measured under two-terminal conFigureuration. The drain voltage was swept in incremental steps from 50 mV to 250 mV for all samples.

**Figure S6.** Cycling stability of transfer characteristics and key device parameters. **(a-c)** Transfer curves measured at intervals of 10 cycles during 50 consecutive cycles for (a) pristine P3HT, (b) P3HT:I_1_, and (c) P3HT:I_1_:I_2_ devices. **(d-f)** Evolution of key device parameters extracted from the transfer curves as a function of cycle number: (d) on/off ratio, (e) threshold voltage (V_TH_), and (f) memory window (ΔV_window_).

**Figure S7.** Cycling stability of transfer characteristics in TT polymer-based ternary devices. **(a-c)** Transfer curves measured at intervals of 10 cycles during 50 consecutive cycles for (a) pristine TT, (b) TT:I_1_, and (c) TT:I_1_:I_2_ devices. **(d-f)** Evolution of key device parameters extracted from the transfer curves as a function of cycle number: (d) on/off ratio, (e) threshold voltage (V_TH_), and (f) memory window (ΔV_window_).

**Figure S8.** Comparison of the operational stability of P3HT, P3HT:I_1_, and P3HT:I_1_:I_2_ devices over 50 consecutive transfer sweeps. (a) Coefficient of variation (CV) of the on/off ratio, (b) threshold-voltage range, and (c) hysteresis-window range. The P3HT:I1:I2 devices exhibit 16.9% of CV, 0.2 V of V_TH_ range, and 0.2 V of V_window_ range Lower values indicate more stable device operation.

**Figure S9.** Device-yield evaluation of P3HT:I_1_:I_2_ devices for assessing operational reproducibility. (a) Three consecutive transfer curves of representative devices D1–D10 measured under identical conditions. (b) Coefficient of variation (CV) of the on/off ratio, (c) threshold-voltage range and (d) hysteresis-window range. The red dashed lines indicate the stability criteria determined from the reference devices in Figure S8.

**Figure S10.** Synaptic plasticity responses under various stimulation conditions. **(a-c)** EPSC responses of pristine P3HT under (a) spike number-, (b) spike amplitude-, and (c) spike width-dependent stimulation. **(d-f)** EPSC responses of P3HT:I_1_ measured under the same conditions as in (a**-**c), demonstrating (d) spike number-, (e) spike amplitude-, and (f) spike width-dependent plasticity. **(g**, **h)** EPSC responses of P3HT:I_1_:I_2_ under (g) spike amplitude- and (h) spike width-dependent stimulation. All pulse conditions were identical to those used in the long-term memory (LTM) measurements, except for the varied stimulation parameter.

**Figure S11.** Universality of ion-exchange-driven ternary blending in DPP-based polymer (TT). **(a-c)** EPSC responses of pristine TT under (a) spike number-, (b) spike amplitude-, and (c) spike width-dependent stimulation. **(d-f)** EPSC responses of TT:I_1_ measured under the same conditions as in (a**-**c), demonstrating (d) spike number-, (e) spike amplitude-, and (f) spike width-dependent plasticity. **(g, h)** EPSC responses of TT:I_1_:I_2_ under (g) spike amplitude- and (h) spike width-dependent stimulation. All pulse conditions were identical to those used in the long-term memory (LTM) measurements, except for the varied stimulation parameter.

**Figure S12.** Long-term memory retention and multi-level storage capability of P3HT:I_1_:I_2_ device. **(a)** Time-resolved EPSC responses of the P3HT:I_1_:I_2_ device measured after applying 50 gate pulses (-3.5 V, 100 ms), demonstrating long-term memory retention over 18 h and 25 h. The EPSC was re-measured following storage under open-circuit conditions, where the gate, source, and drain terminals were electrically disconnected. **(b)** Multi-level storage characteristics obtained by applying 50 consecutive gate pulses (-3.5 V, 100 ms on, 5 s off). The gradual increase in conductance indicates the formation of multiple distinguishable memory states.

 **Figure S13.** Long-term memory retention and multi-level storage capability of TT:I_1_:I_2_ device. **(a)** Time-resolved EPSC responses of the TT:I_1_:I_2_ device measured after applying 50 gate pulses (-3.5 V, 100 ms), demonstrating long-term memory retention over 18 h and 25 h. The EPSC was re-measured following storage under open-circuit conditions, where the gate, source, and drain terminals were electrically disconnected. **(b)** Multi-level storage characteristics obtained by applying 50 consecutive gate pulses (-3.5 V, 100 ms on, 5 s off). The gradual increase in conductance indicates the formation of multiple distinguishable memory states.

**Figure S14.** Experimental setup and device configuration for *in situ* GIWAXS measurements. **(a, b)** photographs of the two-terminal device configuration. A small gap between the Au electrode and the ion-gel was introduced to enable direct exposure of the X-ray beam while allowing controlled electrochemical doping. **(c, d)** Photographs of the *in situ* GIWAXS measurement setup. During the application of 50 gate pulses, the X-ray beam was synchronized to probe the same region multiple times, resulting in a total of 100 GIWAXS measurements at the device gap.

**Figure S15.** 2D GIWAXS patterns and 1D profiles of pristine I_1_ thin film. **(a)** Two-dimensional (2D) GIWAXS pattern of the pristine I_1_ thin film. The I_1_ solution (4 mg mL⁻¹ in chloroform) was spin-coated at 1500 rpm for 40 s onto a silicon wafer. **(b)** One-dimensional (1D) profiles extracted along the out-of-plane (*q*_z_) and in-plane (*q*_xy_) directions.

**Figure S16.** Comparison of out-of-plane 1D GIWAXS profiles of pristine I_1_ and P3HT:I_1_ thin films. Comparison of diffraction peaks for pristine I_1_ (black) and the P3HT:I_1_ binary blend (blue) over different *q_z_* ranges, highlighting the (a) primary (001), (b) secondary (002), and (c) tertiary (003) reflections.

**Figure S17.** Evolution of (100) lamellar diffraction peaks during in situ GIWAXS measurements. Time-resolved evolution of the (100) diffraction peaks obtained from 100 consecutive GIWAXS measurements for (a) P3HT, (b) P3HT:I_1_, and (c) P3HT:I_1_:I_2_. The one-dimensional (1D) profiles extracted along the out-of-plane (*q_z_*) direction are displayed using a color gradient from the first to the final measurement.

**Figure S18.** Gaussian fitting analysis of (100) lamellar diffraction peaks at different pulse states. **(a-c)** (100) diffraction peaks of (a) P3HT, (b) P3HT:I_1_, and (c) P3HT:I_1_:I_2_ at the initial state (0th pulse), fitted using a Gaussian function. **(d**-**f)** Corresponding (100) diffraction peaks of (d) P3HT, (e) P3HT:I_1_, and (f) P3HT:I_1_:I_2_ after electrical stimulation (45th pulse), with Gaussian fitting.

**Figure S19.** Retention stability of (100) lamellar diffraction peaks during in situ GIWAXS measurements. **(a-c)** Time-resolved tracking of the (100) diffraction peaks for (a) P3HT, (b) P3HT:I_1_, and (c) P3HT:I_1_:I_2_ during retention conditions without additional gate pulses. The profiles corresponding to the initial (0th pulse) and doped (45th pulse) states, together with five retention states (retention_1 - retention_5), are plotted. The retention states correspond to the 1st, 25th, 50th, 75th, and 100th GIWAXS measurements acquired during the retention period.

**Figure S20.** Electrochemical impedance spectroscopy and equivalent circuit modeling of P3HT-based devices. **(a)** Nyquist plots of P3HT, P3HT:I_1_, and P3HT:I_1_:I_2_ measured over a frequency range from 1 MHz to 100 mHz. **(b)** Equivalent circuit model used for impedance fitting, based on the metal/ion-gel/semiconductor/electrode device architecture.

**Figure S21.** Nonlinearity (.NL.) fitting using MLP NeuroSim V3 for synaptic weight update characteristics. Fitting of the synaptic nonlinearity (.NL.) curves using the MLP NeuroSim_V3 model at the 50th cycle for (a) P3HT, (b) P3HT:I_1_, and (c) P3HT:I_1_:I_2_ devices.

**Figure S22.** Cycle-dependent evolution of synaptic parameters during LTP and LTD. Key synaptic parameters, including G_max_/G_min_, effective number of states (NS_eff_), and nonlinearity (.NL.), extracted every 10 cycles over 50 LTP/LTD cycles for the P3HT:I_1_:I_2_ device.

**Figure S23.** Stable long-term potentiation and depression under 128 consecutive pulses in P3HT:I_1_:I2 device. **(a)** Endurance characteristics showing 15 cycles of long-term potentiation (LTP) and long-term depression (LTD) under repeated stimulation. All gate pulses were applied at -3.5 V with a pulse interval of 100 ms. **(b)** Evolution of key synaptic parameters, including G_max_/G_min_, effective number of states (NS_eff_), and nonlinearity (.NL.), extracted every 3 cycles.

**Note S1. Calculation detail**

Ab initio molecular dynamics (AIMD) simulations were performed within the framework of density functional theory (DFT) using the Vienna Ab initio Simulation (VASP) Package.^[58, 59]^ The electron–ion interactions were described using the Projector Augmented Wave Method. ^[60]^ and the exchange–correlation functional was treated within the Generalized Gradient Approximation using the Perdew–Burke–Ernzerhof Functional (GGA-PBE). ^[61]^ A plane-wave cutoff energy of 600 eV was used. Prior to the AIMD simulations, the structures were fully relaxed until the residual forces on each atom were smaller than 0.01 eV Å⁻¹.

The AIMD simulations were carried out in the canonical (NVT) ensemble using a Nose–Hoover thermostat. ^[62, 63]^ with a time step of 1 fs for several picoseconds. The Brillouin zone was sampled at the Γ-point due to the large supercell size. The resulting trajectories were analyzed to investigate ion dynamics and structural evolution.

**Note S2. MNIST classification detail**

To accurately capture the synaptic device behavior within the neural framework, a linear mapping was implemented between the hardware-aware CNN model weights [W_min_, W_max_] and the experimentally measured conductance [G_min_, G_max_] for the device. The forward mapping is defined as:

$G=G_{min}+\left( W-W_{min} \right) \frac{G_{max}-G_{min}}{W_{max}-W_{min}}$…………………….…….… [1]

while the inverse mapping, applied during gradient-based weight updates, is expressed as:

$W=W_{min}+\left( G-G_{min} \right) \frac{W_{max}-W_{min}}{G_{max}-G_{min}}$…………………….……… [2]

Training was performed using the categorical cross-entropy loss function:

$\mathcal{L=}-\sum_{i=1}^{c} y_{i}\log\left( \hat{y}_{i} \right)$…………………………….…. [3]

where *C* is the total number of classes, *y_i_* is the true class label, and $\hat{y}_{i}$ is the predicted probability for class *i*. For each epoch, the forward pass produced network predictions and corresponding loss, while backpropagation computed gradients *∂L*/*∂W*. Conductance updates were then applied based on the sign and magnitude of the gradient:

$\Delta G\propto-\eta\frac{\partial L}{\partial W}$……………………………………. [4]

and then quantized to the nearest valid potentiation or depression level derived from the device's measured neuromorphic behavior.

**Reference**

[40]	Shan, W., Wang, Y., Zhong, Y., Wustoni, S., Inal, S., "Organic Electrochemical Synaptic Transistors with Improved Retention for Logic and Biosignal Processing," *Advanced Materials* 38, no. 4 (2026).

[41]	Woo, G. W., Lee, C. M., Lee, W. W., et al., "Detachable and Reusable: Reinforced π‐Ion Film for Modular Synaptic Reservoir Computing," *Advanced Materials* 37, no. 41 (2025).

[42]	Won, Y., Lee, J., Kim, H., Oh, J. H., "Enhancing Synaptic Plasticity and Multistate Retention of Organic Neuromorphic Devices Using Anion‐Excessive Gel Electrolyte," *Advanced Functional Materials* 36, no. 26 (2026).

[43]	Lee, D., Li, M. Q., An, M., Sung, J., Lee, J., Lee, E., "Fundamental Control of Cation–Anion Interactions Governing Retentive Behavior in Organic Synaptic Transistor," *ACS nano* 19, no. 51 (2025).

[44]	Kim, N., Go, G.-T., Park, H.-L., et al., "Molecular tailoring to achieve long‐term plasticity in organic synaptic transistors for neuromorphic computing," *Advanced Intelligent Systems* 5, no. 9 (2023).

[45]	Lee, M., Shin, D., Cheon, J., et al., "Ferroelectric–Electrolyte Hybrid Gate Dielectrics for Organic Synaptic Transistors with Long‐Term Plasticity," *Advanced Electronic Materials* (2026).

[46]	Lee, D., Kim, M., Park, S., et al., "Inter‐Ion Mutual Repulsion Control for Nonvolatile Artificial Synapse," *Advanced Functional Materials* 35, no. 11 (2025).

[47]	Wang, L., Yin, X., Cheng, H., et al., "Improving Nonvolatile Properties of Solid‐Electrolyte‐Based Artificial Synapses via Ion Dynamics Modulation in Organic Electrochemical Transistors," *SmartMat* 6, no. 4 (2025).

[58]	Kresse, G., Furthmüller, J., "Efficiency of ab-initio total energy calculations for metals and semiconductors using a plane-wave basis set," *Computational materials science* 6, no. 1 (1996).

[59]	Kresse, G., Furthmüller, J., "Efficient iterative schemes for ab initio total-energy calculations using a plane-wave basis set," *Physical review B* 54, no. 16 (1996).

[60]	Blöchl, P. E., "Projector augmented-wave method," *Physical review B* 50, no. 24 (1994).

[61]	Perdew, J. P., Burke, K., Ernzerhof, M., "Generalized gradient approximation made simple," *Physical review letters* 77, no. 18 (1996).

[62]	Nosé, S., "A unified formulation of the constant temperature molecular dynamics methods," *The Journal of chemical physics* 81, no. 1 (1984).

[63]	Hoover, W. G., "Canonical dynamics: Equilibrium phase-space distributions," *Physical review A* 31, no. 3 (1985).
